# Bacterial efflux pump modulators prevent bacterial growth in macrophages and under broth conditions that mimic the host environment

**DOI:** 10.1128/mbio.02492-23

**Published:** 2023-11-03

**Authors:** Samual C. Allgood, Chih-Chia Su, Amy L. Crooks, Christian T. Meyer, Bojun Zhou, Meredith D. Betterton, Michael R. Barbachyn, Edward W. Yu, Corrella S. Detweiler

**Affiliations:** 1 Molecular, Cellular, and Developmental Biology, University of Colorado Boulder, Boulder, Colorado, USA; 2 Department of Pharmacology, Case Western Reserve University School of Medicine, Cleveland, Ohio, USA; 3 Cleveland Center for Membrane and Structural Biology, Case Western Reserve University School of Medicine, Cleveland, Ohio, USA; 4 Chemical and Biological Engineering, University of Colorado Boulder, Boulder, Colorado, USA; 5 Duet Biosystems, Nashville, Tennessee, USA; 6 Antimicrobial Research Consortium (ARC) Labs, Boulder, Colorado, USA; 7 Department of Physics, University of Colorado, Boulder, Colorado, USA; 8 Center for Computational Biology, Flatiron Institute, New York, New York, USA; 9 Barbachyn Consulting, LLC, Kalamazoo, Michigan, USA; University of Utah, Salt Lake City, Utah, USA

**Keywords:** AcrAB-TolC, cryo-electron microscopy, Gram-negative bacteria, efflux pump, macrophage, SAFIRE

## Abstract

New approaches for combating microbial infections are needed. One strategy for disrupting pathogenesis involves developing compounds that interfere with bacterial virulence. A critical molecular determinant of virulence for Gram-negative bacteria are efflux pumps of the resistance-nodulation-division family, which includes AcrAB-TolC. We previously identified small molecules that bind AcrB, inhibit AcrAB-TolC, and do not appear to damage membranes. These efflux pump modulators (EPMs) were discovered in an in-cell screening platform called SAFIRE (Screen for Anti-infectives using Fluorescence microscopy of IntracellulaR Enterobacteriaceae). SAFIRE identifies compounds that disrupt the growth of a Gram-negative human pathogen, *Salmonella enterica* serotype Typhimurium (*S*. Typhimurium), in macrophages. We used medicinal chemistry to iteratively design ~200 EPM35 analogs and test them for activity in SAFIRE, generating compounds with nanomolar potency. Analogs were demonstrated to bind AcrB in a substrate binding pocket by cryo-electron microscopy. Despite having amphipathic structures, the EPM analogs do not disrupt membrane voltage, as monitored by FtsZ localization to the cell septum. The EPM analogs had little effect on bacterial growth in standard Mueller Hinton Broth. However, under broth conditions that mimic the micro-environment of the macrophage phagosome, *acrAB* is required for growth, the EPM analogs are bacteriostatic, and the EPM analogs increase the potency of antibiotics. These data suggest that under macrophage-like conditions, the EPM analogs prevent the export of a toxic bacterial metabolite(s) through AcrAB-TolC. Thus, compounds that bind AcrB could disrupt infection by specifically interfering with the export of bacterial toxic metabolites, host defense factors, and/or antibiotics.

**IMPORTANCE:**

Bacterial efflux pumps are critical for resistance to antibiotics and for virulence. We previously identified small molecules that inhibit efflux pumps (efflux pump modulators, EPMs) and prevent pathogen replication in host cells. Here, we used medicinal chemistry to increase the activity of the EPMs against pathogens in cells into the nanomolar range. We show by cryo-electron microscopy that these EPMs bind an efflux pump subunit. In broth culture, the EPMs increase the potency (activity), but not the efficacy (maximum effect), of antibiotics. We also found that bacterial exposure to the EPMs appear to enable the accumulation of a toxic metabolite that would otherwise be exported by efflux pumps. Thus, inhibitors of bacterial efflux pumps could interfere with infection not only by potentiating antibiotics, but also by allowing toxic waste products to accumulate within bacteria, providing an explanation for why efflux pumps are needed for virulence in the absence of antibiotics.

## INTRODUCTION

Infections caused by Gram-negative bacteria are particularly challenging to treat because their cell envelope incorporates complementary defenses that protect them from chemicals (1–3). The outer membrane has an external leaflet that is relatively impermeable to chemicals because it consists of tightly packed, negatively charged lipopolysaccharide (LPS) molecules bound to magnesium cations, which together repel hydrophobic molecules (4). The outer membrane surrounds a porous cell wall and the inner membrane. Compounds that manage to breach the outer membrane are typically captured in the periplasm or inner membrane by efflux pumps and are exported across both membranes. Thus, antibiotics that slowly permeate the outer membrane are prevented from accumulating to critical levels within the bacterium (2, 5, 6).

Select efflux pumps are demonstrated virulence determinants, meaning they are required for bacteria to cause infection even in the absence of treatment with antibiotics (7–10). This observation could reflect that efflux pumps are needed to expel metabolic waste and/or host defense molecules during infection. Nevertheless, these same efflux pumps are not essential for bacterial growth in standard broth culture (6). In patients treated with commonly used antibiotics, bacterial efflux pumps typically contribute to bacterial multidrug resistance (MDR) by capturing and exporting the antibiotics, increasing the probability of treatment failure and the emergence of resistant clones (11). For these reasons, there is interest in developing inhibitors of efflux pumps that could re-sensitize MDR bacteria to antibiotics and/or prevent pathogen export of toxic metabolites and host defense molecules (6, 12).

In *Escherichia coli* and the closely related *Salmonella enterica* serotype Typhimurium (*S*. Typhimurium), the major efflux pump required for virulence is AcrAB-TolC, a member of the classic resistance-nodulation-division (RND) family. AcrAB-TolC is a multi-subunit machine that spans the inner membrane, periplasm, and outer membrane (13). In the inner membrane, AcrB assembles into a trimer and traps substrates from the lipid bilayer or the periplasm. The AcrB trimer has a large substrate binding pocket that can manage substrates with diverse structures. AcrB transfers substrates to the periplasmic subunit of the pump, AcrA, which in turn passes substrates to the TolC channel in the outer membrane, enabling extrusion out of the cell (14–17).

We previously described three small molecules that inhibit AcrAB-TolC and were called efflux pump modulators (EPMs): EPM30, EPM35, and EPM43. These EPMs were discovered in a screen for compounds that prevent *S*. Typhimurium from colonizing macrophages in a screening platform called SAFIRE (Screen for Anti-infectives using Fluorescence microscopy of IntracellulaR Enterobacteriaceae) (18, 19). In the test tube*,* the EPMs bind AcrB, and in bacteria, they interfere with the export of substrates by AcrAB-TolC. In macrophages, the EPMs synergize with antibiotics that are exported by AcrAB-TolC, including ciprofloxacin, erythromycin, and chloramphenicol, suggesting they target AcrAB-TolC during infection (13, 18, 20, 21). The EPMs may also target other efflux pumps, as they synergize with cationic antimicrobial peptides not known to be AcrAB-TolC substrates in *E. coli,* such as LL-37 and polymyxin B (18, 22, 23). Moreover, the EPMs inhibit the survival of both wild-type and *acrAB-*deficient *S*. Typhimurium in macrophages and in HeLa cells. The EPMs did not have apparent deleterious effects on bacterial or mammalian membranes, a key characteristic because dissipation of proton motive force deactivates efflux pumps non-specifically by depriving them of energy. However, these three EPMs disrupted mammalian cell morphology, indicating toxicity (18). We therefore embarked on an iterative approach that combined medicinal chemistry with activity screening in SAFIRE to improve EPM potency and decrease toxicity.

## RESULTS

### Design and screening of EPM analogs

**Fig 1:**
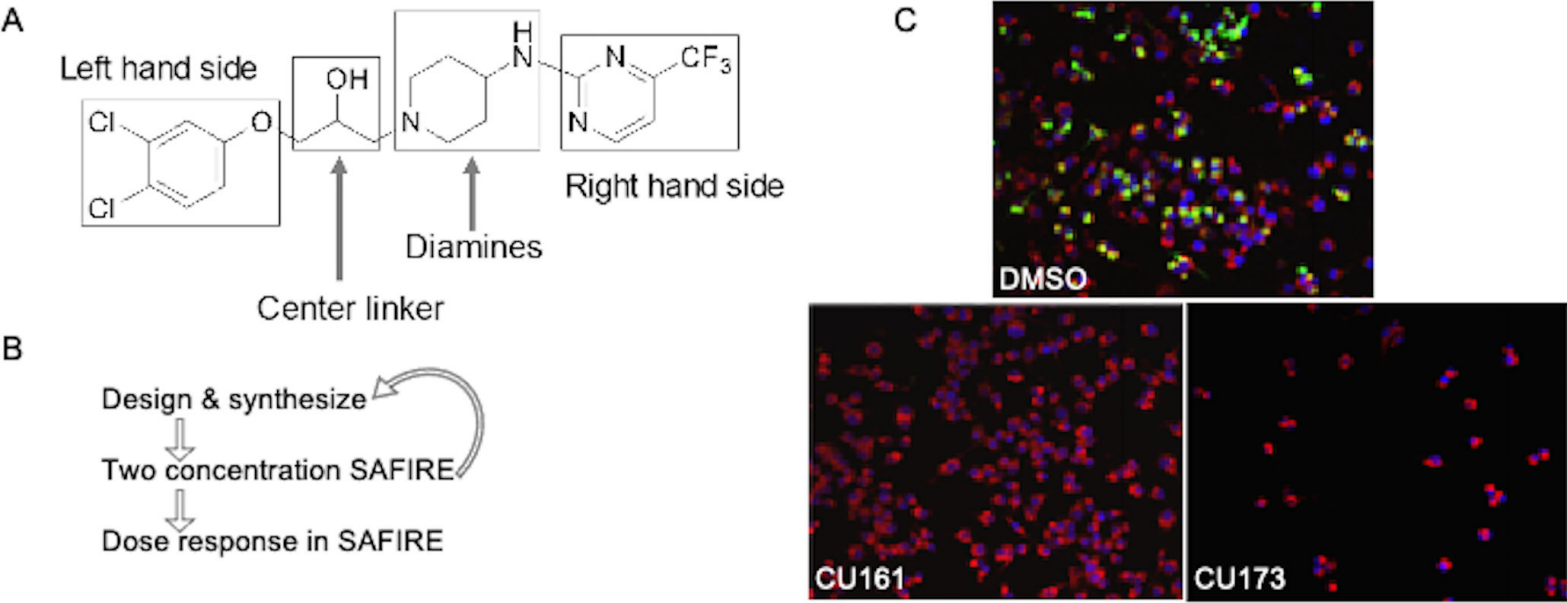
Approach for the improvement of EPM activity in the SAFIRE assay and reduction of toxicity. (**A**) Structural components of EPM35 modified by medicinal chemistry. (**B**) Iterative screening pathway. The two concentrations tested in SAFIRE were 5 µM and 25 µM. Dose responses were performed with at least eight analog concentrations. (**C**) Examples of infected macrophages treated with control dimethylsulfoxide (DMSO), a non-toxic (CU161), and a toxic (CU173) analog, as evidenced by the small number of macrophages apparent 16 h after treatment. *sifB::gfp* (green), MitoTracker (red), 4′,6-diamidino-2-phenylindole (blue).

All three of the EPMs previously identified were used to inform early structure-activity relationship efforts because their activity profiles suggested distinct efflux modulation profiles (18). However, the three EPMs share related chemical motifs. For example, EPM30 contains a guanidine moiety, which is chemically similar to the aminopyrimidine moiety in EPM35 and the diamino-benzopyrimidine moiety in EPM43. We designed and synthesized analogs in batches of 20–50, up to approximately 200 chemical compounds. Our initial synthetic efforts primarily focused on varying the left-hand side, the right-hand side, and the diamine moieties of EPM35, with modifications to the 2-hydroxypropanyl central linker limited to simple O-alkylation and preparation of enantiomeric pairs to explore the effect of absolute configuration on activity (Fig. 1A). On the left-hand side, we examined analogs containing substitutions of the phenyl ring with, for instance, methoxy, alkyl, cyano, or trifluoromethyl groups, halogens, and several combinations thereof. On the right-hand side, we experimented with removing the 6-trifluoropyrimidin-2-yl group, consistent with another goal of the medicinal chemistry campaign, which was to identify the minimum pharmacophore for this series of compounds. We also altered the diamine subunits with (i) the introduction of N-alkyl, N,N-dialkyl, and N-acetyl groups in place of the simple primary amine, as well as (ii) investigating a variety of isosteric cyclic amine ring systems, for example, pyrrolidine, to replace the starting piperidine ring of EPM35. Each of the 200 analogs was vetted by liquid chromatography–mass spectrometry (LC-MS) for purity >95% and by proton nuclear magnetic resonance (^1^H NMR) for the intended structure.

**Fig 2:**
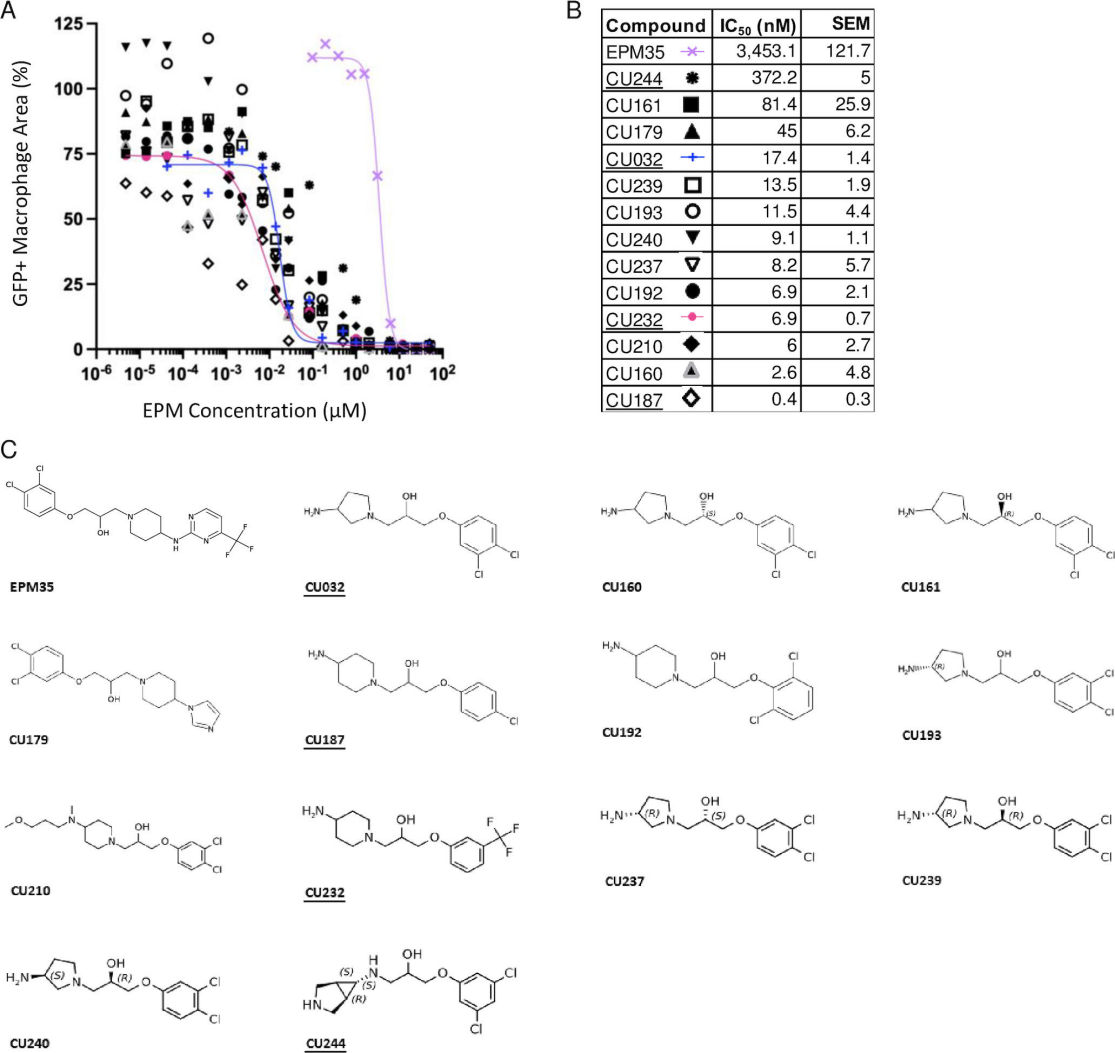
SAFIRE IC_50_ curves, values, and structures of EPM35 (parent compound) and the top 13 EPM analogs. (**A and B**) IC_50_ curves and values. Data are derived from >10 concentrations per analog and are normalized to DMSO-treated control samples (100%). (**C**) Structures. EPM analogs examined in subsequent figures are underlined.

Analogs were iteratively tested for potency and toxicity at two concentrations, 5 µM and 25 µM, in the SAFIRE macrophage assay (Fig. 1B). In SAFIRE, we used MATLAB software to count the number of macrophages that remain adherent and retain the red fluorescence of MitoTracker, an indicator of voltage across the mitochondrial inner membrane. MATLAB then calculates the signal from bacteria (green fluorescent protein [GFP]) that overlaps with macrophages, as defined by MitoTracker, to estimate bacterial load. This process revealed that substitutions on the amine nitrogen compromised antibacterial activity in SAFIRE (Table S1). If a compound reduced the percentage of adherent macrophages to 60% or less than DMSO-controls or resulted in poor macrophage morphology after 16 h of exposure, then we eliminated the compound as host-toxic (Fig. 1C; Table S1). For example, we observed that the 6-trifluoropyrimidin-2-yl group on the right-hand side of EPM35 is apparently toxic to macrophages. Non-toxic analogs that reduced bacterial load by at least 60% at 5 µM and 25 µM in SAFIRE, as compared with DMSO, progressed and were evaluated in SAFIRE across eight or more concentrations, ranging from 0.001 µM to 50 µM (*n* = 2). Calculated half maximal inhibitory concentration (IC_50_) values suggest that in-cell antibacterial activity increased more than 100-fold for some of the analogs compared to the parent compound EPM35 (Fig. 2). Each of the top 13 analogs had at least an eightfold increase in potency and were not toxic for macrophages at 50 µM in the SAFIRE assay (Fig. 1C).

### The EPM analogs interact with AcrB

We selected three compounds for further study, CU032, CU232, and CU244, because they incorporated different aspects of the parent structure, specifically the negative atoms on the benzene ring and the amine on opposite side of the molecule. To determine whether the EPMs could bind AcrB, we used purified *E. coli* AcrB, which is 95% identical to *S. enterica* Typhimurium AcrB. We previously used isothermal titration calorimetry (ITC) (18, 24) to show that AcrB interacts with the EPM35 parent compound with a K_D_ in the sub-micromolar range (18). Here, we show with ITC that, like EPM35, the three analogs are capable of specifically interacting with AcrB to form complexes (Fig. S1; Table 1). Curiously, the analogs have K_D_s that are 25- to 50-fold larger than that of EPM35. In addition, the binding of CU032 and CU232 to AcrB releases significantly less enthalpy (H) and increases entropy (S), indicating that these two EPM analogs rely on the entropic effect for binding, whereas binding of the parent EPM35 ligand to AcrB is more dependent on the enthalpic effect. Nevertheless, our ITC data revealed equilibrium dissociation constants (K_D_s) for AcrB and the EPM analogs that are in good agreement with those of AcrB substrates: K_D_s of AcrB with the EPM analogs range from 7.4 µM to 15 µM, whereas K_D_s of AcrB with rhodamine 6G, proflavine, and ciprofloxacin range from 5.5 µM to 74.1 µM (25). Thus, the EPM analogs interact specifically with AcrB in a manner consistent with established EPM substrates.

**TABLE 1:** Binding of EPMs to AcrB, as calculated from ITC results

Compound	K_D_ (µM)	∆H cal/mol	∆S cal/(mol deg)
EPM35^*a*^	0.29 ± 0.03	−7,663 ± 84	4.2
CU032	14.7 ± 9.56	−1,947 ± 490	15.60
CU232	7.41 ± 1.00	−2,124 ± 93	16.30
CU244	15.0 ± 3.81	−8,654 ± 1,384	−6.91

^*a*^ EPM35 was previously reported (18) and is included here for comparison.

### EPM analogs bind to AcrB in the substrate hydrophobic pocket

**Fig 3:**
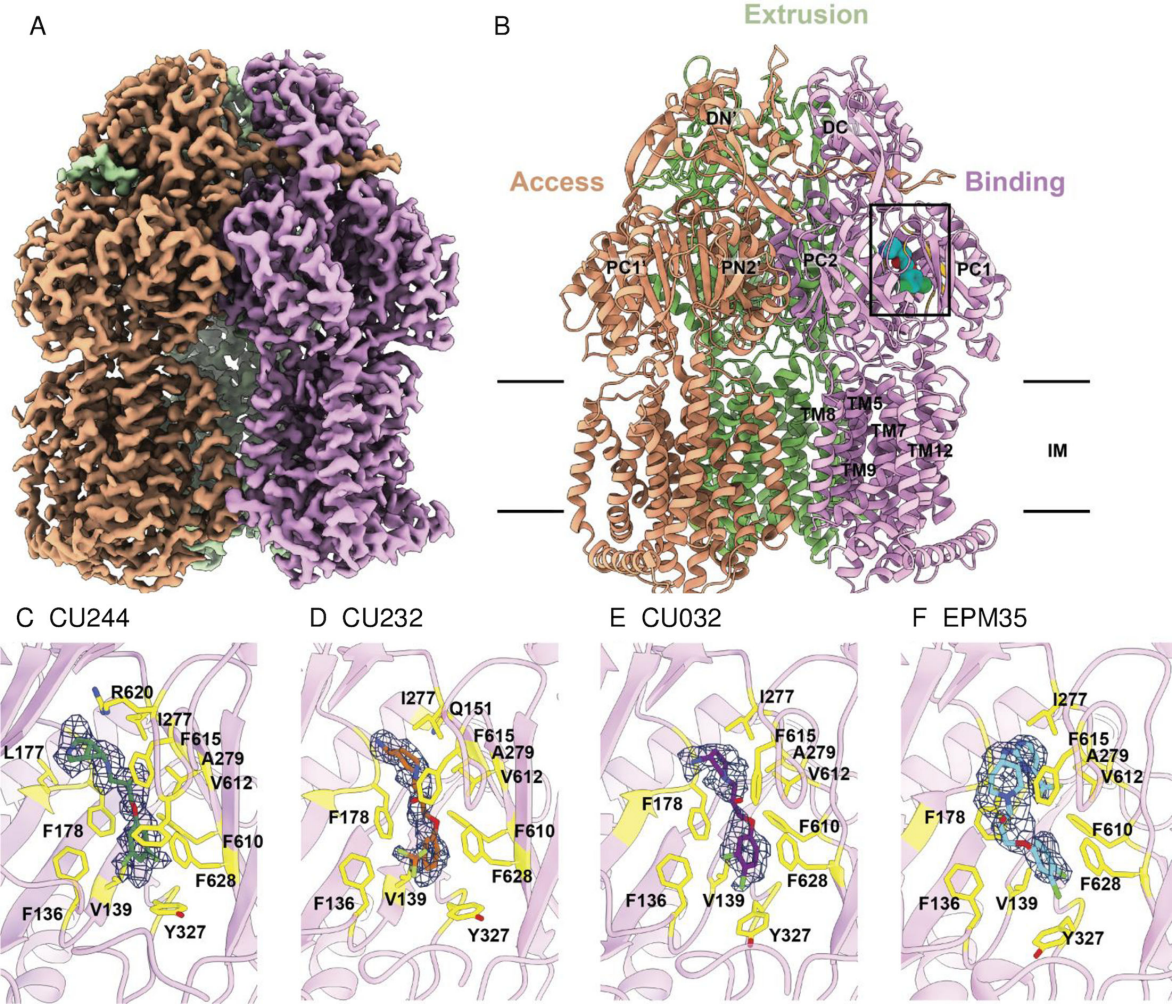
Single-particle cryo-EM structure of the AcrB-analog complexes. (**A**) Cryo-EM density map of trimeric AcrB-CU244. (**B**) Ribbon diagram of the structure of the side view of the AcrB-CU244 trimer. The bound CU244 molecule within the “binding” protomer of AcrB-CU244 is represented by cyan spheres. In (**A and B**), the “access,” “extrusion,” and “binding” protomers are colored orange, green, and pink, respectively. Each protomer of AcrB contains 12 transmembrane helices (TM1–TM12) and six periplasmic subdomains (PN1, PN2, PC1, PC2, DN, and DC). (**C**) The CU244 binding site. The bound CU244 molecule is colored green. (**D**) The CU232 binding site. The bound CU232 molecule is colored orange. (**E**) The CU032 binding site. The bound CU032 molecule is colored purple. (**F**) The CU035 binding site. The bound EPM35 molecule is colored cyan. In (**C–F**), residues participating in compound binding are in yellow sticks. The cryo-EM densities of bound compounds are colored dark blue.

To elucidate at high resolution how the EPM analogs interact with AcrB, we used cryo-electron microscopy (cryo-EM). We overproduced and purified the *E. coli* AcrB protein containing a 6×His tag at the C-terminus and reconstituted the protein into lipidic nanodiscs (17). We incubated the AcrB-nanodisc samples with the parent compound or with one of three structurally dissimilar EPMs analogs (CU032, CU232, CU244) to form AcrB-EPM complexes. We then solved single-particle cryo-EM structures of these complexes to resolutions between 2.63 and 2.82 Å (Table 2; Fig. 3).

**TABLE 2:** Cryo-EM data collection, processing, and refinement statistics

Data set	CU32	EPM35	CU232	CU244
Data collection and processing				
Magnification		81,000 300 Krios-GIF-K3 −1.0 to −2.5 3.8 20 1.07 36 38 16		
Voltage (kV)				
Electron microscope				
Defocus range (μm)				
Total exposure time (s)				
Energy filter width (eV)				
Pixel size (Å)				
Total dose (e^-^/Å^2^)				
Number of frames				
Dose rate (e^-^/phys. pixel/s)				
No. of initial micrographs	2,683	2,229	1,267	1,002
No. of initial particles	1,680,995	3,244,867	1,270,215	998,124
No. of final particles	237,148	155,685	161,598	162,5048
Symmetry	C1	C1	C1	C1
Resolution (Å)	2.62	2.71	2.71	2.44
FSC threshold^*b*^	0.143	0.143	0.143	0.143
Resolution range	2.39–6.79	2.50–9.80	2.40–7.15	2.39–9.84
Refinement				
Model resolution cut-off (Å)	2.62	2.71	2.71	2.44
Model composition				
No. of protein residues	3,099	3,099	3,099	3,099
RMSD^*a*^				
Bond lengths (Å)	0.003	0.003	0.003	0.004
Bond angles (°)	0.554	0.746	0.554	0.658
Validation				
MolProbity score	1.13	1.42	1.13	1.22
Clash score	3.42	4.20	3.42	2.05
Ramachandran plot (%)				
Favored (%)	98.16	96.57	98.16	99.03
Allowed (%)	1.84	3.43	1.84	0.97
Disallowed (%)	0	0	0	0
CC mask	0.82	0.81	0.82	0.83
CC box	0.66	0.65	0.64	0.65
CC vol	0.78	0.78	0.78	0.81

^*a*^ Root mean square deviation.

^*b*^ Fourier shell correlation.

#### Structure of AcrB-CU244

The single-particle images of the AcrB-CU244 complex led to a high-quality cryo-EM map, allowing us to solve the structure at a resolution of 2.44 Å (Table 2; Fig. S2). CU244 is housed in a large cavity created by the distal drug-binding site of the “binding” protomer of AcrB, where the periplasmic cleft is completely open. There are at least 23 amino acids involved in forming the distal drug-binding site of AcrB (26, 27). In addition, this distal site includes a hydrophobic trap that contributes strongly to drug binding (27). This trap includes residues F178, I277, V612, and F615. Within 4 Å of bound CU244, there are 12 residues, including F136, V139, L177, F178, I277, A279, Y327, F610, V612, F615, R620, and F628, involved in anchoring this EPM (Fig. 3C). Most of these residues are hydrophobic in nature, suggesting that the binding is mainly governed by hydrophobic and aromatic interactions. Surprisingly, no hydrogen bonds are found to facilitate CU244 binding, highlighting the importance of these hydrophobic and aromatic residues for substrate recognition.

#### Structure of AcrB-CU232

We incubated the AcrB-nanodisc sample with CU232 to form the AcrB-CU232 complex and solved this complex structure using single-particle cryo-EM. The reconstituted sample led to a cryo-EM map at a nominal resolution of 2.71 Å (Table 2; Fig. S3). Superimposition of the AcrB-CU244 and AcrB-CU232 trimers gives rise to a calculated root mean square deviation (RMSD) of 0.5 Å, indicating that the conformations of these two trimers are nearly identical to each other. Again, CU232 is only observed to bind within the “binding” protomer, whereas no ligands are seen in the “access” and “extrusion” protomers. In the “binding” protomer of AcrB-CU232, CU232 is also found within the distal drug-binding site. Amino acids within 4 Å of bound CU232 include F136, V139, Q151, F178, I277, A279, Y327, F610, V612, F615, and F628 (Fig. 3D). Most of these 11 residues are hydrophobic and aromatic in nature, underscoring that this EPM analog recognition is largely governed by hydrophobic and aromatic interactions.

#### Structure of AcrB-CU032

We also produced the AcrB-CU032 complex by incubating AcrB-nanodisc with CU032. The reconstituted sample led to a high-quality cryo-EM map, permitting us to solve the cryo-EM structure of the AcrB-CU032 complex to a resolution of 2.62 Å (Table 2; Fig. S4). As with CU244 and CU232, the bound CU032 molecule is only found within the “binding” protomer, leaving the drug-binding sites of both the “access” and “extrusion” protomers unoccupied. The binding mode of CU032 within the “binding” protomer is similar to those of CU244 and CU232, where CU032 is anchored in the distal drug-binding pocket. However, it appears that AcrB utilizes a slightly different subset of residues to secure this modulator. Within 4 Å of bound CU032, residues F136, V139, F178, I277, A279, Y327, F610, V612, F615, and F628 participate in anchoring this ligand (Fig. 3E). All 10 residues are hydrophobic and aromatic in nature, denoting that modulator binding is mostly controlled by hydrophobic and aromatic interactions.

#### Structure of AcrB-EPM35

We also assembled the AcrB-EPM35 complex and solved its cryo-EM structure. The single-particle images led to a cryo-EM map at nominal resolution of 2.71 Å (Table 2; Fig. S5). Like AcrB-CU244, AcrB-CU232, and AcrB-CU032, the trimeric AcrB-EPM35 complex is asymmetric in conformation and displays the “access,” “binding,” and “extrusion” protomer structures. Pairwise superimpositions of the AcrB-CU244, AcrB-CU232, AcrB-CU032, and AcrB-EPM35 trimer provide RMSD values between 0.4 and 0.6 Å, suggesting that the structures of these four trimers are nearly identical to each other. The bound EPM35 molecule is only found within the “binding” protomer of AcrB, where its binding mode is very similar to those of CU244, CU232, and CU032. EPM35 largely resides at the distal drug-binding pocket of the periplasmic domain of the “binding” protomer. Within 4 Å of bound EPM35, residues F136, V139, F178, I277, A279, Y327, F610, V612, F615, and F628 are responsible for anchoring this modulator (Fig. 3F). These 10 residues are identical to those involved in CU032 binding. Again, it is observed that AcrB contacts EPM35 via hydrophobic and aromatic interactions. No hydrogen bonds or significant electrostatic interactions are found to facilitate the binding.

### Proton motive force is not dissipated by the EPM analogs

**Fig 4:**
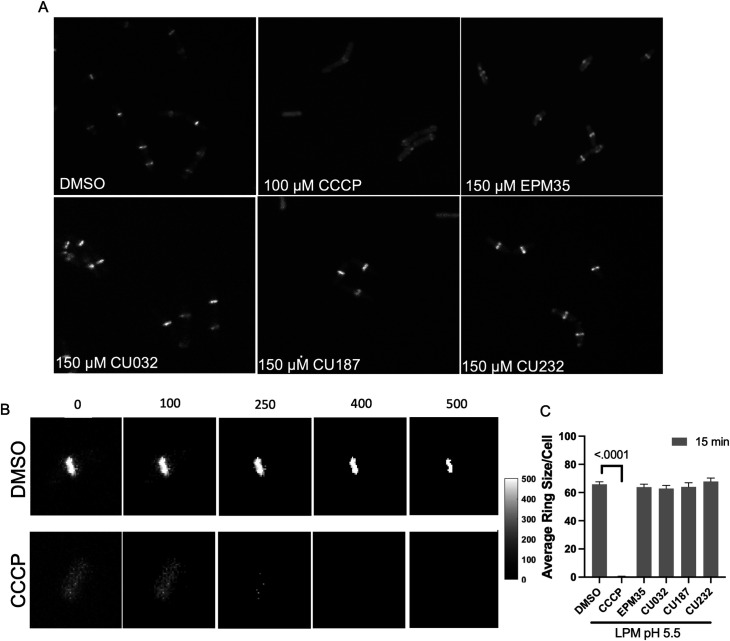
The EPM analogs minimally disrupt membrane voltage. (**A**) Micrographs of *Bacillus subtilis* expressing mNeongreen-FtsZ were grown to mid log-phase in low phosphate, low magnesium medium (LPM) 5.5. Samples were pipetted onto LPM 5.5 agar pads containing either DMSO or with 150 µM of EPM35 or the EPM analog indicated for 15 min and imaged by confocal fluorescent microscopy. (**B**) How FtsZ rings were quantified: overall intensity levels per pixel were quantified and a threshold of 400 was set, above which, signal was measured to establish the FtsZ ring size within the cell. Examples of thresholds are shown. (**C**) Cells exposed to EPM35 or EPM analogs were imaged at 15 after treatment, as indicated. Y-axis is the average ring size in pixels per cell. For CCCP, the ring size was on average one pixel per cell. At least 50 cells from three biological replicates were compared for each sample. Error bars are SEM. *P*-value of <0.05 compared to DMSO calculated with a one-way analysis of variance and a Tukey-Kramer post-test.

The structural data suggest that the EPM analogs could be substrates of efflux pumps. However, the EPM analogs could also indirectly interfere with pump activity: their amphipathic nature suggests they could interact with membranes and damage the inner membrane barrier, which efflux pumps require for energy. We therefore monitored membrane integrity in the presence of EPM analogs by evaluating membrane voltage with mNeongreen-FtsZ (28–30). The FtsZ protein localizes to the bacterial septum based on membrane potential, and rapid delocalization of fluorescent FtsZ reporters occurs when voltage across the membrane is disrupted (31, 32). We monitored mNeongreen-FtsZ localization in a Gram-positive bacterium, *Bacillus subtilis,* to eliminate the potentially confounding issue of the passage of EPM analogs through the outer membrane at different rates (24, 33–36). As expected, treatment of *B. subtilis* cells with the protonophore carbonyl cyanide m-chlorophenyl hydrazone (CCCP) (100 µM) delocalized FtsZ (Fig. 4) (31). We determined that CU032 and CU232, two of the compounds examined with AcrB by cryo-EM, had no significant effect on the size of the mNeongreen-FtsZ ring after 15 min of treatment at 150 µM. Likewise, CU187, the most active EPM analog in SAFIRE, and the EPM35 parent compound did not alter FtsZ localization. These data suggest that the EPM analogs do not indirectly inhibit efflux pumps by damaging membranes, dissipating proton motive force, and thereby generally depleting energy.

### AcrAB-TolC likely exports a toxic bacterial metabolite under broth conditions that mimic the phagosome microenvironment

**Fig 5:**
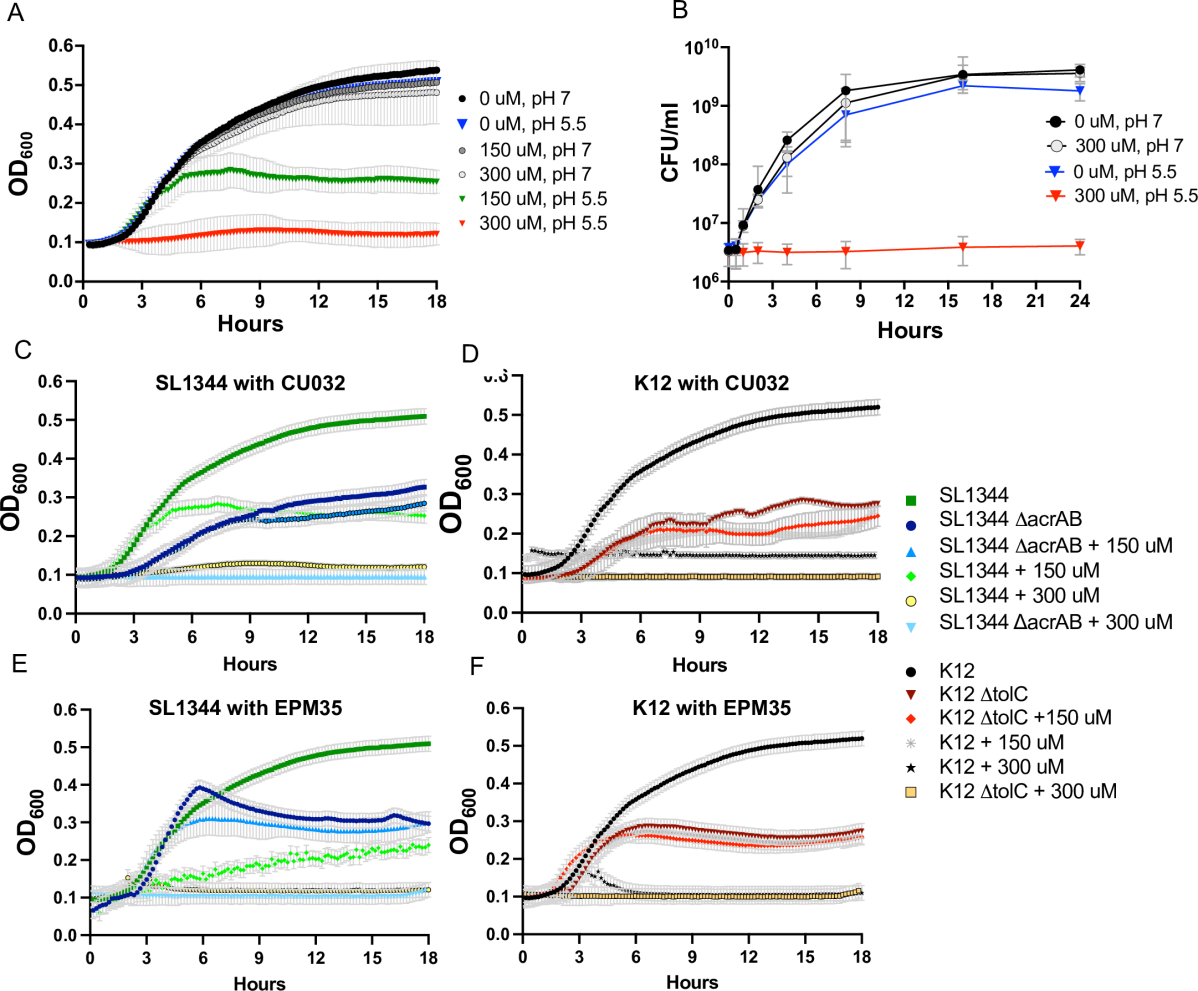
In LPM pH 5.5, *acrAB* and *tolC* are required for growth, and EPM analogs inhibit growth. (**A and B**) Optical density at 600 nm (OD_600_) and CFU per milliliter of *S*. Typhimurium in LPM 5.5 (at pH 5.5) or LPM 7 (at pH 7.0) in the presence of CU032. (C through F) OD_600_ in LPM 5.5 of (C and **E**) *S*. Typhimurium *ΔacrAB* mutant and wild-type strains, and (**D and F**) *E. coli ΔtolC* mutant and wild-type strains with (**C, D**) CU032 or (**E and F**) EPM35. Mean and SEM of three biological replicates are shown for A–F. The wild-type and mutant strain controls are the same for C and E and for D and F.

Given that the EPM analogs appear to directly inhibit AcrAB-TolC activity, we examined whether they interact with antibiotics to inhibit growth in media. Checkerboard assays are typically performed in Mueller Hinton Broth (MHB) (37, 38), but in this medium CU032 did not inhibit the growth of wild-type bacteria (Fig. S6), prohibiting a full analysis of EPM-antibiotic interactions. However, CU032 prevented bacterial growth in low phosphate, low magnesium medium (LPM) 5.5, a medium that mimics the microenvironment of the macrophage phagosome and can result in a leaky outer membrane (34) (Fig. 5A). LPM 5.5 is chemically defined, contains low phosphate and low magnesium, and is buffered to pH 5.5 (34, 39–41). Changing the pH of this medium to 7.0 abolished the CU032 inhibitory activity, which was bacteriostatic, not bactericidal (Fig. 5B). It was further noted that mutant strains lacking *acrAB* or *tolC* grow poorly in LPM 5.5 compared to the corresponding wild type (Fig. 5C through F). These observations suggest that in LPM 5.5, *S*. Typhimurium utilizes AcrAB-TolC to export a growth inhibitory metabolite. The addition of CU032 or EPM35 to the medium of the *ΔacrAB* or *ΔtolC* mutant strains does not strongly inhibit growth compared to DMSO, suggesting that neither compound has a significant target beyond efflux pumps. In sum, the data support that LPM 5.5 is an appropriate medium for quantifying EPM-antibiotic interactions and show that in this medium, *S*. Typhimurium appears to accumulate an unknown, bacteriostatic metabolite(s) that is normally expelled by AcrAB-TolC.

### EPM analogs enhance the potency of ciprofloxacin and erythromycin

**Fig 6:**
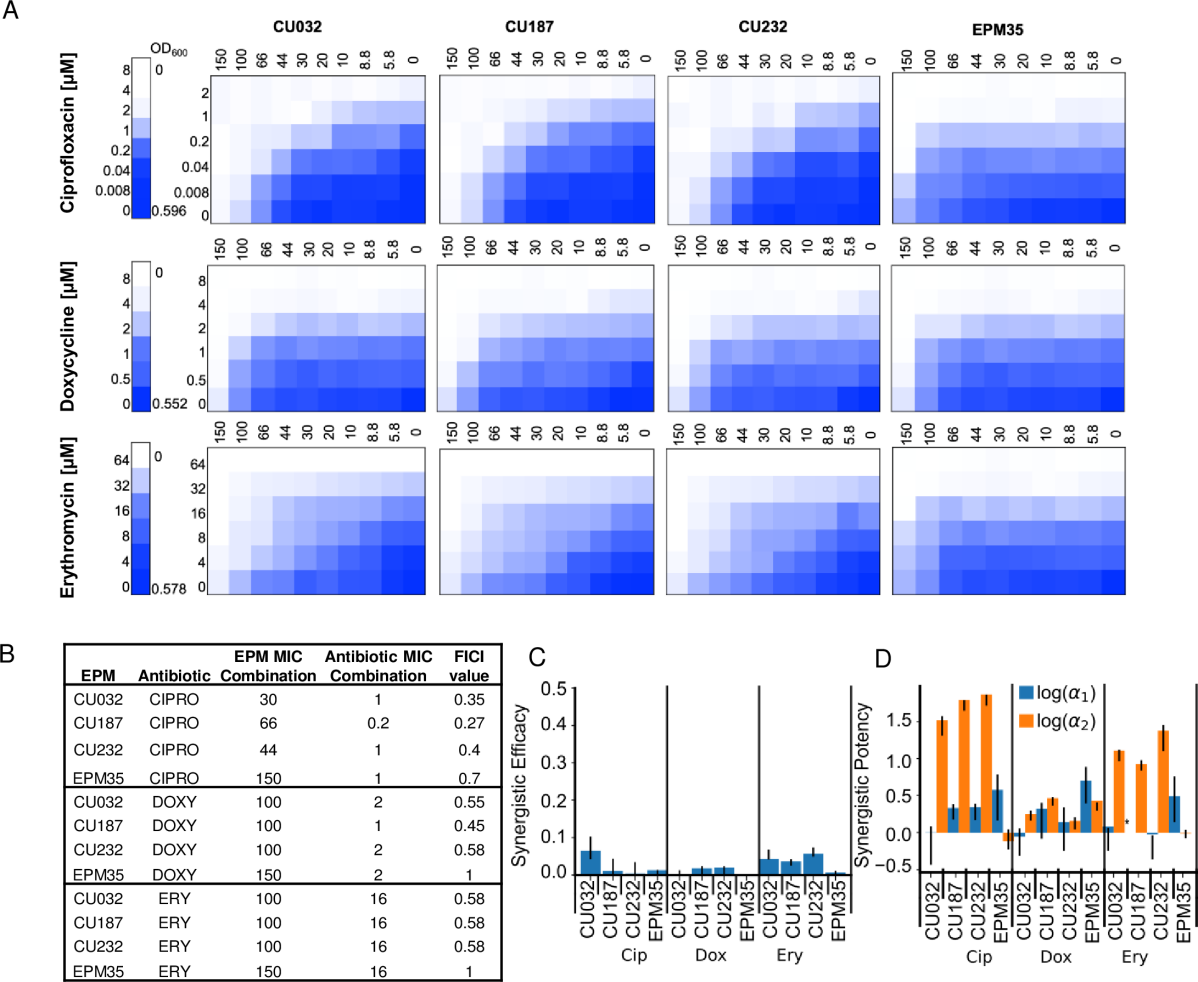
EPM analogs, but not the parent EPM35 compound, increase the potency of ciprofloxacin and erythromycin. (**A**) Checkerboard assays with *S*. Typhimurium and three classes of clinical antibiotics. Data are means of at least three biological replicates. (**B**) FICI calculations. (**C and D**) MuSyC comparisons. (**C**) β_abs_ values demonstrate little effect of EPM35 or the EPM analogs on antibiotic efficacy. (**D**) Alpha1 (blue) represents the effect of the antibiotic on the compound, and alpha 2 (orange) represents the effect of the compound on the antibiotic.

To examine whether the EPM analogs interact additively or synergistically with antibiotics, checkerboard growth assays were carried out in LPM 5.5. We compared three antibiotics (ciprofloxacin, doxycycline, erythromycin) and three EPM analogs (CU032, CU187, CU232) and the EPM35 parent compound (Fig. 6A). Standard fractional inhibitory concentration index (FICI) analyses supported synergy between the EPM analogs and ciprofloxacin (Fig. 6B). Since FICI values cannot distinguish between synergy due to changes in potency (activity) versus efficacy (the extent of the effect) (42, 43), we used the MuSyC synergy algorithm to measure EPM-antibiotic synergy (44, 45). The data were well fit by the MuSyC equation, with an *R*^2^ of greater than 0.96 for all checkerboards (Table S2). Combining each of the EPM analogs with any one of the three antibiotics did not increase the maximal observed antibiotic efficacy by more than 7% (β_obs ≤ 0.07, Fig. 6C; Table S2). Instead, all three EPMs increased the potency of each antibiotic by up to 72-fold [log_10_(α2) ≤1.87] and the increase was largest for ciprofloxacin, followed by erythromycin, then doxycycline (Fig. 6D; Table S2). In contrast, the antibiotics did not increase the potency of the EPM analogs by more than twofold [log(α1) <0.4]. The EPM35 parent compound did not synergize with the antibiotics, indicating that the SAFIRE-based screening process identified compounds that are better at inhibiting efflux pumps than EPM35. Moreover, the consistency in the synergy of potency values across the antibiotics with different EPM analogs points toward a shared mechanism of action: the EPMs could modify the pharmacodynamics of the efflux process and prevent export of specific substrates through interactions with AcrB. We posit that the EPMs differentially reduce antibiotic export resulting in a higher effective intracellular concentration of the antibiotic.

## DISCUSSION

We set out to increase the potency and decrease the toxicity of a series previously described EPMs (18). Iterative medicinal chemistry and screening in the SAFIRE cell culture assay identified EPMs with 1,000-fold increases in activity, with toxicity below 50 µM. ITC and cryo-EM studies demonstrated that the EPM analogs specifically bind the AcrB inner membrane protein of the AcrAB-TolC efflux pump. The ITC-derived K_D_ of the EPM analogs was approximately 20-fold higher than that of the parent compound. However, all of these AcrB and EPM binding interactions are within the micromolar range, consistent with the binding affinities of AcrB with a variety of compounds (25). For instance, the *Klebsiella pneumoniae* AcrB multidrug efflux pump, which shares 91% protein sequence identity with AcrB from *E. coli*, distinctively contacts the antibiotic erythromycin with a K_D_ value of 14.4 µM ± 2.6 µM (46). This binding affinity is in good agreement with the strength of AcrB-EPM interactions revealed in this work. Within AcrB, the analogs occupy the hydrophobic substrate binding pocket, which is large and captures diverse molecules (13, 16, 25, 47). The EPMs interact with multiple amino acid residues in the pocket through hydrophobic bonds: no hydrogen bonding nor salt bridges were observed despite having the resolution required to detect them. These observations reveal that the EPMs may interfere with pump function in a manner that disrupts infection.

Despite their amphipathic structures, the EPMs do not appear to disrupt membranes. This is an important observation because dissipation of the proton motive force starves the cell for energy and indirectly reduces efflux. Delocalization of the septal protein FtsZ is a rapid and sensitive assay for proton motive force (PMF) disruption (31, 32). We used a *B. subtilis* model system for this assay to avoid issues of compound exclusion from the cell membrane by an outer membrane barrier, an approach that could be useful for establishing whether other apparent EPMs damage membranes. While we worked with a MNeongreen labeled FtsZ protein for this purpose (48), other proteins also localize to the bacterial cell septum or membrane because they have a short alpha-helical region that associates with charged membranes (49, 50). Thus, a number of reporters (32) could be used to determine whether inhibitors of efflux adversely affect the proton gradient.

Efflux pumps are virulence determinants; they are not required for bacterial replication in standard laboratory media but are needed during infection in mammalian cells and/or animals (6, 51). In accordance with these prior observations, inhibitors of efflux pumps, including the EPM parent compounds, their analogs, and a series of promising pyranopyridines, do not prevent bacterial growth in standard microbiological media, specifically cation-adjusted MHB and/or lysogeny broth (LB) (18, 52, 53). We found that in LPM 5.5 medium, *acrAB* and *tolC* are required for *S*. Typhimurium and *E. coli* growth, respectively. LPM 5.5 was designed to mimic the acidified microenvironment of the macrophage phagosome (39–41) and potentiates the activity of antimicrobials (34, 54). Bacterial growth in LPM 5.5 could require *acrAB* and *tolC* because a bacteriostatic toxic metabolite(s) needs to be expelled by efflux pumps in this medium. This same potential metabolite(s) could accumulate in bacteria treated with the EPM analogs in LPM 5.5, explaining why the analogs inhibit growth under these conditions. For these reasons, LPM 5.5 medium could provide researchers with a future opportunity to identify an endogenous substrate(s) of AcrAB-TolC of relevance to the role of efflux pumps in virulence.

The medium LPM 5.5 mimics the macrophage phagosome in that it is acidic and has low levels of phosphate and magnesium. The latter are needed for outer membrane integrity: magnesium stabilizes LPS (55, 56), and phosphate plays a key structural role in LPS glycosidic linkages (57–60). Bacteria grown in LPM 5.5 have demonstrated increased outer membrane permeability compared to bacteria grown in standard media (34, 54). In LPM 5.5, the growth inhibitory activity of the EPM analogs could reflect that small molecules have improved access to AcrB and therefore prevent the export of a toxic metabolite at lower concentrations. Similarly, EPMs could potentiate antibiotics in LPM 5.5 due to improved antibiotic access to the bacterial cell. LB medium, like LPM 5.5, is deficient in divalent cations (61–63), suggesting that pyranopyridine efflux pump inhibitors act synergistically with antibiotics in LB (47) due to leakage of antibiotics and/or of the pump inhibitors across the outer membranes. Conditions within the macrophage phagosome also damage Gram-negative bacterial outer membranes, facilitate antibiotic access, and expose the pathogen to host-derived antimicrobials (64–66). Therefore, the EPMs could be particularly effective at reducing bacterial load in macrophages compared to in LPM 5.5 due to a combination of the accumulation of putative toxic bacterial metabolites and/or of host cell-autonomous defenses.

The use of the MuSyC synergy algorithm to analyze the checkerboard data from growth in LPM 5.5 enabled deeper probing of EPM-antibiotic interactions than standard FICI analyses (42, 43). MuSyC fits checkerboard data to a 10-parameter, two-dimensional Hill equation. It requires a Hill-like dose–response curve for each single compound and distinguishes between synergy resulting from increases in potency, efficacy, and/or cooperativity (44, 45). MuSyC revealed that the interaction of the EPM analogs and antibiotics occurs directionally: the EPMs increase the effective concentration of the antibiotics (synergy of potency), but the reverse does not occur. Furthermore, the EPMs do not increase the maximal extent to which antibiotics prevent growth (efficacy), instead, they reduce the dose at which antibiotics inhibit growth (potency). The antibiotics in turn do not enhance the ability of the EPMs to prevent the export of a potential toxic metabolite(s). These observations support a causal relationship between the biochemical (i.e., AcrB-binding) and phenotypic activity (i.e., growth inhibition) of the EPMs. In other words, EPM binding to AcrB appears to directly interfere with the activity of the efflux pump.

Limitations of these studies include that LPM 5.5 does not fully mimic the macrophage phagosome microenvironment, which is dynamic and includes antimicrobial peptides, lysozyme, complement, and additional host antimicrobials and innate immune defenses recruited from vesicles and the cytosol (67–69). In addition, the EPM parent compounds or analogs could have unknown off-target activities. Data indicating that the parent compounds have bacterial targets beyond AcrB include the observation that they reduce *ΔacrAB* mutant *S*. Typhimurium accumulation in macrophages below that of DMSO-treated controls (18). Possible alternative targets for the EPMs in macrophages include the multiple *acrB* orthologs encoded by Enterobacteriaceae (7, 14). It is also conceivable that in macrophages, the parent compounds or EPM analogs have a mammalian target(s). We conclude that the 1,000-fold increased activity of the EPM analogs in the SAFIRE macrophage assay relative to the parent EPMs could be due to a combination of factors, including increased access to bacteria caused by a weakened outer membrane barrier, the accumulation within intracellular bacteria of toxic efflux pump substrates, lysosomal trapping (35, 70), and possibly undefined off-target effects.

In summary, iterations of medicinal chemistry and a series of unconventional assays, including SAFIRE, cryo-EM, FtsZ localization, and checkerboards in LPM 5.5 medium, have improved upon and further characterized a family of EPMs. Specifically, these approaches enabled the generation of a set of related compounds that bind AcrB and appear to target efflux pumps in their capacity as virulence determinants, by preventing the export of an unknown toxic metabolite(s).

## MATERIALS AND METHODS

### Bacterial strains and media

We used *S. enterica* subspecies *enterica* serovar Typhimurium strain SL1344 (71), *S*. Typhimurium strain *ΔacrAB* (ALR1257) in the SL1344 background (18), *S*. Typhimurium strain 14028s (ATCC), and *S*. Typhimurium strain S10801 (NR-22067), a multidrug-resistant isolate from a calf with sepsis (72) obtained through Biodefense and Emerging Infections Research Resources Repository (BEI resources), National Institute of Allergy and Infectious Diseases (NIAID), and National Institutes of Health.

Two *E. coli* strains were used, K-12 derivative BW25113 (wild type) (73) and K-12 *ΔtolC* [JP313 *ΔtolC* (74), also called AD3644 and JLD1285]. *Bacillus subtilis* strain DK5092 (mNeonGreen-FtsZ) was constructed as described previously (28, 75).

Bacteria were grown in LB at 37°C (76, 77), unless otherwise stated. Cation-adjusted MHB was purchased from Sigma-Aldrich (90922). LPM 5.5 medium was made according to the following recipe: 5 mM KCl, 7.5 mM (NH_4_)2SO_4_, 0.5 mM K_2_SO_4_, 10 mM glucose, 49 µM MgCl_2_, 337 µM PO_4_-, 0.05% casamino acids (CAS)-amino acids, 80 mM MES, adjusted to pH to 5.5 with 5 M NaOH, and sterile filtered. Where stated, bacteria were grown with antibiotics: 30 µg/mL streptomycin.

### SAFIRE screening for potency and toxicity

The initial screening of compounds in SAFIRE was performed at two concentrations, 5 µM and 25 µM, followed by dose–response curves with a minimum of eight concentrations per compound to determine the IC_50_. As previously described (18), RAW 264.7 macrophages (between passages 1 and 6) were grown in complete Dulbecco’s modified Eagle’s medium (DMEM) to approximately 70% to 90% confluence, scraped, washed, resuspended, diluted to 5 × 10^4^ in 100 µL, and seeded in black, 96-well, glass-bottomed plates (Brooks Life Sciences catalog number MGB096-1-2-LG-L) at 37°C with 5% CO_2_. Twenty-four hours later, bacteria grown overnight in LB were diluted into 50 µL phosphate buffered saline (PBS) and added to a final concentration of 1 × 10^7^ *S*. Typhimurium SL1344 (*sifB::gfp*) per milliliter (78) for an approximate multiplicity of infection of 30 bacteria per macrophage cell. Forty-five minutes after bacterial addition, gentamicin (Sigma catalog number G1264) was added to a final concentration of 40 µg/mL to inhibit the growth of remaining extracellular bacteria. Two hours after infection, gentamicin-containing medium was removed and replaced with 200 µL fresh DMEM with compound or DMSO (Sigma catalog number 276855) to the stated final concentrations. At 17.5 h after infection, PBS containing MitoTracker Red CMXRos (Life Technologies catalog number M7512), a vital dye for mitochondrial electric potential, was added to a final concentration of 100 nM. At 18 h after infection, 16% paraformaldehyde was added to a final concentration of 4% and incubated at room temperature for 15 min. Cells were washed twice with PBS and stained for 20 min with 1 µM 4′,6-diamidino-2-phenylindole (DAPI), a vital dye for double-stranded DNA, and stored in 90% glycerol in PBS until imaging. Samples were imaged in six fields of view per well using a semiautomated Yokogawa CellVoyager CV1000 confocal scanner system with a 20×, 0.75-numerical aperture (NA) objective. A MATLAB algorithm calculated bacterial accumulation (GFP fluorescence) within macrophages, as defined by DAPI and MitoTracker Red. GFP^+^ macrophage area was defined as the number of GFP-positive pixels per macrophage divided by the total number of pixels per macrophage, averaged across all macrophages in the field (34).

### ITC

AcrB protein was purified as described (25). Briefly, the AcrB protein contains a 4×His tag at the C-terminus and was overproduced in *E. coli* BL21-Gold (DE3) cells (Stratagene) using the plasmid derived from pSPORT1 (Invitrogen) (79). Cells were grown in 6 L of LB medium with 100 µg/mL ampicillin and disrupted with a French pressure cell. The membrane fraction was collected and washed twice with buffer containing 20 mM sodium phosphate (pH 7.2), 2 M KCl, 10% glycerol, 1 mM EDTA, and 1 mM phenylmethanesulfonyl fluoride (PMSF), and once with 20 mM HEPES–NaOH buffer (pH 7.5) containing 1 mM PMSF. The membrane proteins were then solubilized in 1% (wt/vol) n-dodecyl β-D-maltoside (DDM). Insoluble material was removed by ultracentrifugation at 370,000 × *g*. The extracted protein was purified with Cu^2+^-affinity and G-200 sizing columns (13, 80). The purified AcrB protein was then concentrated to a final monomeric concentration of 10 µM in buffer containing 20 mM Na-HEPES (pH 7.5) and 0.05% DDM. Similar protein purification procedures were extensively used to elucidate structure-function of RND transporters, including AcrB (13, 16, 25), CusA (81, 82), MtrD (83, 84), CmeB (85), AdeB (86), HpnN (87), and MmpL3 (88). We also used these protein purification protocols for *in vitro* substrate transport study via the CusA transporter (82) and *in vitro* functional dynamics measurement of the CmeB transporter (85), indicating that these purified membrane proteins are fully functional *in vitro*.

Briefly, measurements were performed on a Microcal iTC200 (Malvern Panalytical) at 25°C. Before titration, the protein was dialyzed against buffer containing 20 mM Na-HEPES (pH 7.5), 0.05% n-dodecyl-µ-maltoside (DDM), and 5% DMSO (18). The Bradford assay was used to quantify protein concentration, which was adjusted to a final monomeric concentration of 10 µM. Ligand solution consisting of 100 µM EPM in the aforementioned buffer was prepared as the titrant. Both the protein and ligand samples were degassed before loading the samples. Two-microliter injections of the ligand were used for data collection. Injections occurred at intervals of 60 s and lasted for 4 s. Heat transfer (µcal/s) was measured as a function of elapsed time (s). The mean enthalpies measured from injection of the ligand in the buffer were subtracted from raw titration data before data analysis with ORIGIN software (MicroCal). Titration curves fitted with a non-linear regression fitting to the binding isotherm provided the equilibrium binding constant (*K*_A_ = 1/*K*_D_) and enthalpy of binding (Δ*H*). Based on the values of *K*_A_, the change in free energy (Δ*G*) and entropy (Δ*S*) were calculated with the equation Δ*G* = *−RT* ln*K*_A_ = Δ*H − T*Δ*S*, where *T* is 2/3 *K* and *R* is 1.9872 cal/K per mol. Calorimetry trials were also carried out in the absence of AcrB using the same experimental conditions. No change in heat was observed in the injections throughout the experiment.

### Cryo-EM

AcrB was purified from *E. coli* BL21(DE3)*ΔacrB*/pSPORTΩ*acrB* cells and reconstituted into lipidic nanodiscs. To assemble AcrB into nanodiscs, a mixture containing 10 µM AcrB, 30 µM membrane scaffold protein (1E3D1), and 900 µM *E. coli* total extract lipid was incubated for 15 min at room temperature. After, 0.8 mg/mL prewashed Bio-Beads (Bio-Rad) was added. The resultant mixture was incubated for 1 h on ice, followed by overnight incubation at 4°C. The protein-nanodisc solution was filtered through 0.22 µm nitrocellulose filter tubes to remove the Bio-Beads. To separate free nanodiscs from AcrB-loaded nanodiscs, the filtered protein-nanodisc solution was purified using a Superose six column (GE Healthcare) equilibrated with 20 mM Tris-HCl (pH 7.5) and 100 mM NaCl. Fractions corresponding to the size of the trimeric AcrB-nanodisc complex were collected for cryo-EM (46). We then incubated 0.6 mg/mL AcrB-nanodisc with 10 µM EPM for 2 h and solved structure of the AcrB-EPM complex using single-particle cryo-EM to a resolution of 2.20 Å.

### Membrane voltage disruption assay (FtsZ delocalization)

To determine whether exposure to compound dislodges FtsZ from the cell septum, an overnight culture of *Bacillus subtilis* grown in LPM at pH 5.5 (LPM 5.5) expressing mNeonGreen-FtsZ (strain DK5092) was exposed to DMSO, CCCP (100 µM) or an EPM analog (150 µM) for 15 or 30 min, as indicated. Bacteria were immobilized on 1% agarose pads made with LPM under a coverslip. To set the threshold value for the FtsZ septal ring, mNeonGreen signal (excitation 488 nm, emission 525 nm) from at least 20 DMSO-treated cells was quantified using a CV1000 confocal microscope with a 100× oil, NA = 1.4 objective. A background-subtracted image was made by subtracting the median intensity from full original images. A signal threshold of ≥400 arbitrary units per pixel was set (Fig. 4B). Then, we manually confirmed that mNeonGreen signal above threshold was at the center of the bacterial rod. We examined 50 cells per sample from at least three biological replicates (≥150 cells per condition). A MATLAB program (https://github.com/Betterton-Lab/FtsZ-Quantification) counted the number of pixels above threshold per cell and averaged this value across all cells for each condition and this value is reported in the graph (Fig. 4C).

### Checkerboard assays and analyses

Overnight cation-adjusted MHB-grown cultures of SL1344 or SA10801 were diluted in MHB or LPM 5.5 to an optical density at 600 nm (OD_600_) of 0.01 and distributed into polystyrene 96-well flat-bottom plates. EPM analogs were added at concentrations up to 300 µM, near the limit of solubility. The antibiotics ciprofloxacin, doxycycline, and erythromycin were added up to concentrations of 2 µM, 2 µM, and 8 µM, respectively. The final DMSO concentration was at or below 2% in all wells. Plates were grown at 37°C with shaking for 18 h and OD_600_ was monitored (BioTek Synergy H1, BioTek Eon). Each experiment was performed with biological triplicate or higher as indicated. Percent growth was determined by normalizing to OD_600_ reads in wells containing no D66 or antibiotic. Checkerboards in LPM 5.5 medium were performed with culture grown overnight in LB and diluted to an OD_600_ of 0.01 in LPM 5.5 and compounds/antibiotics were added as previously described. MuSyC analysis was pursued as previously described (44, 45). Briefly, the 2D Hill equation was fit to the normalized growth inhibition data from the checkerboards using a bounded non-linear least square regression algorithm. A Monte Carlo sampling method was used to compute the 95% CI in the parameter fits from three biological replicates. The bounds on the parameters minimal and maximal efficacy of the antibiotics and EPMs (E_0_,E_max_) were [0.99,1.01] and [0,1], respectively (44, 45).

## SUPPLEMENTAL MATERIAL

Supplemental Table and Figures mbio.02492-23-s0001.docx Table S2 and Fig. S1 to FS6.

Table S1 mbio.02492-23-s0002.xlsx Analog testing in SAFIRE.

ASM does not own the copyrights to Supplemental Material that may be linked to, or accessed through, an article. The authors have granted ASM a non-exclusive, world-wide license to publish the Supplemental Material files. Please contact the corresponding author directly for reuse.
