# Supplementary material for: Bacterial efflux pump modulators prevent bacterial growth in macrophages and under broth conditions that mimic the host environment
Source: mBio. 2023 Nov 3;14(6):e02492-23. doi: 10.1128/mbio.02492-23 (PMC10746280; doi:10.1128/mbio.02492-23)
Supplement: Supplemental Table and Figures — Table S2 and Fig. S1 to FS6. [file mbio.02492-23-s0001.docx]

## SUPPLEMENTAL MATERIAL

## Bacterial Efflux Pump Modulators Prevent Bacterial Growth in Macrophages and Under Broth Conditions that Mimic the Host Environment

Samual C. Allgood^1*^, Chih-Chia Su^2,3*^, Amy L. Crooks^1^, Christian T. Meyer^1,4-6^, Bojun Zhou^7^, Meredith D. Betterton^1,7,8^, Michael R. Barbachyn^9^, Edward W. Yu^2,3^, Corrella S. Detweiler^1^

* These authors contributed equally to the manuscript.

^1^ Molecular, Cellular Developmental Biology, University of Colorado Boulder, Boulder, CO, USA

^2^ Department of Pharmacology, Case Western Reserve University School of Medicine, Cleveland, OH 44106, USA

^3^ Cleveland Center for Membrane and Structural Biology, Case Western Reserve University School of Medicine, Cleveland, OH 44106, USA

^4^ Chemical and Biological Engineering, University of Colorado Boulder, Boulder, CO, USA

^5^ Duet Biosystems, Nashville, TN, USA

^6^ Antimicrobial Research Consortium (ARC) Labs, Boulder, CO, USA

^7^ Department of Physics, University of Colorado, Boulder, CO, USA

^8^ Center for Computational Biology, Flatiron Institute, New York, NY, USA

^9^ Barbachyn Consulting, LLC, Kalamazoo, MI, USA

Correspondence: detweile@colorado.edu, ewy5@case.edu

**Table S1 Analog Testing in SAFIRE (see downloadable xls file)**

**Table S2. MuSyC synergy fit values.**

| Antibiotic | EPM | Fit R^2^ | Β_obs_ [95% CI]^1^ | log_10_(α1) [95% CI] ^2^ | log_10_(α2) [95% CI] ^3^ |
| --- | --- | --- | --- | --- | --- |
| Ciprofloxacin | CU032 | 0.96 | 0.07 [0.05,0.1 ] | 0.0 [-0.42,0.07] | 1.52 [1.32,1.56] |
|  | CU187 | 0.98 | 0.01 [-0.01,0.04] | 0.33 [0.19,0.37] | 1.79 [1.66,1.78] |
|  | CU232 | 0.98 | 0.0 [-0.02,0.03] | 0.34 [0.18,0.38] | 1.87 [1.73,1.85] |
|  | EPM35 | 0.98 | 0.01 [-0.01,0.01] | 0.58 [0.17,0.77] | -0.12 [-0.22,0.03] |
| Doxycycline | CU032 | 0.98 | 0.0 [-0.01,0.01] | -0.06 [-0.3,0.04] | 0.25 [0.17,0.28] |
|  | CU187 | 0.97 | 0.02 [-0.0,0.02] | 0.32 [-0.07,0.39] | 0.46 [0.37,0.47] |
|  | CU232 | 0.97 | 0.02 [-0.01,0.02] | 0.14 [-0.23,0.33] | 0.16 [0.06,0.19] |
|  | EPM35 | 0.98 | -0.01 [-0.04,-0.01] | 0.7 [0.41,0.87] | 0.43 [0.31,0.39] |
| Erythromycin | CU032 | 0.98 | 0.04 [0.04,0.07] | 0.08 [-0.23,0.05] | 1.11 [0.98,1.11] |
|  | CU187 | 0.99 | 0.04 [0.03,0.04] | nan [-4.0,-0.82] | 0.93 [0.85,0.96] |
|  | CU232 | 0.98 | 0.06 [0.05,0.07] | -0.02 [-0.35,-0.05] | 1.38 [1.11,1.44] |
|  | EPM35 | 0.99 | 0.01 [-0.01,0.01] | 0.49 [0.15,0.74] | -0.01 [-0.06,0.03] |

^1^ Β_obs_ is the proportion increase in maximal efficacy of the antibiotics at maximum tested dose of EPMs.

^2^ log_10_(α1) is the log-fold-change in potency of EPMs due to the antibiotics. Shown in brackets are the 95% confidence interval (CI) for the MuSyC fits.

^3^ log_10_(α2) is the log-fold-change in potency of the antibiotics due to the EPMs.

**Figure S1**

**
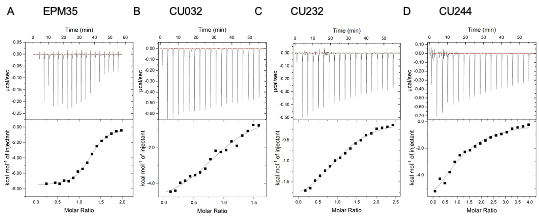
**

**Fig S1. Binding of the EPM analogs to AcrB as shown by ITC.**

**A - D)** Representative ITC for the binding of EPM35 and three analogs to AcrB. Each peak in the upper panel corresponds to the injection of 2 μL of 100 μM of EPM in buffer containing 20 mM Na-HEPES (pH7.5), 0.05% DDM and 5% DMSO into the reaction containing 10 μM of monomeric AcrB in the same buffer. The lower panel shows the cumulative heat of reaction displayed as a function of injection number. The solid line is the least-square fit to the experimental data.


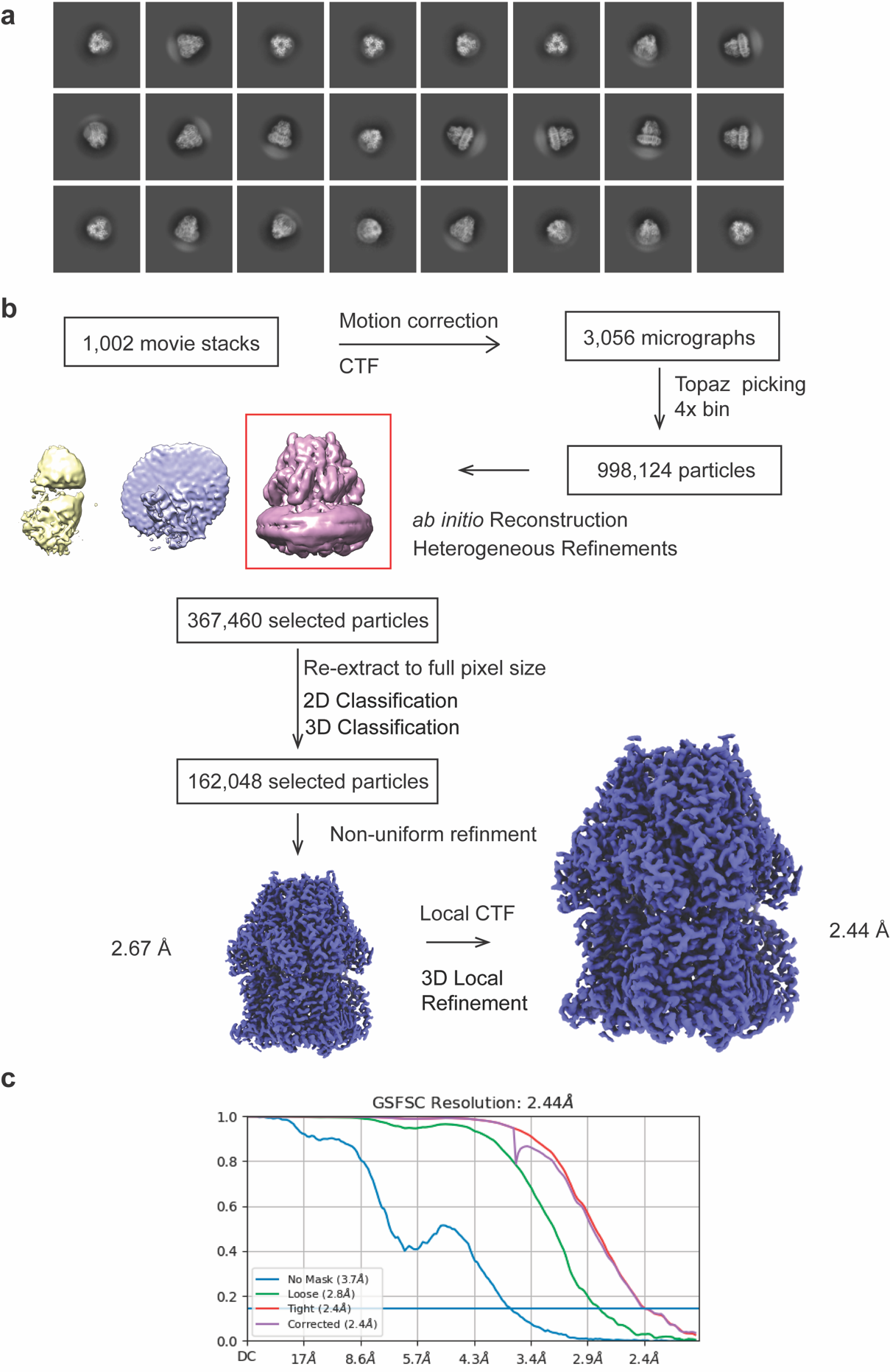
**Figure S2**

**Figure S2. AcrB-CU244 data processing.**  **A)** Representative 2D classes of AcrB-CU244. **B)** Data processing workflow of AcrB-CU244. The side view density map of AcrB-CU244 is colored blue. (c) Gold-Standard Fourier shell correlation (GS-FSC) curves of AcrB-CU244, showing final resolution of 2.44 Å.


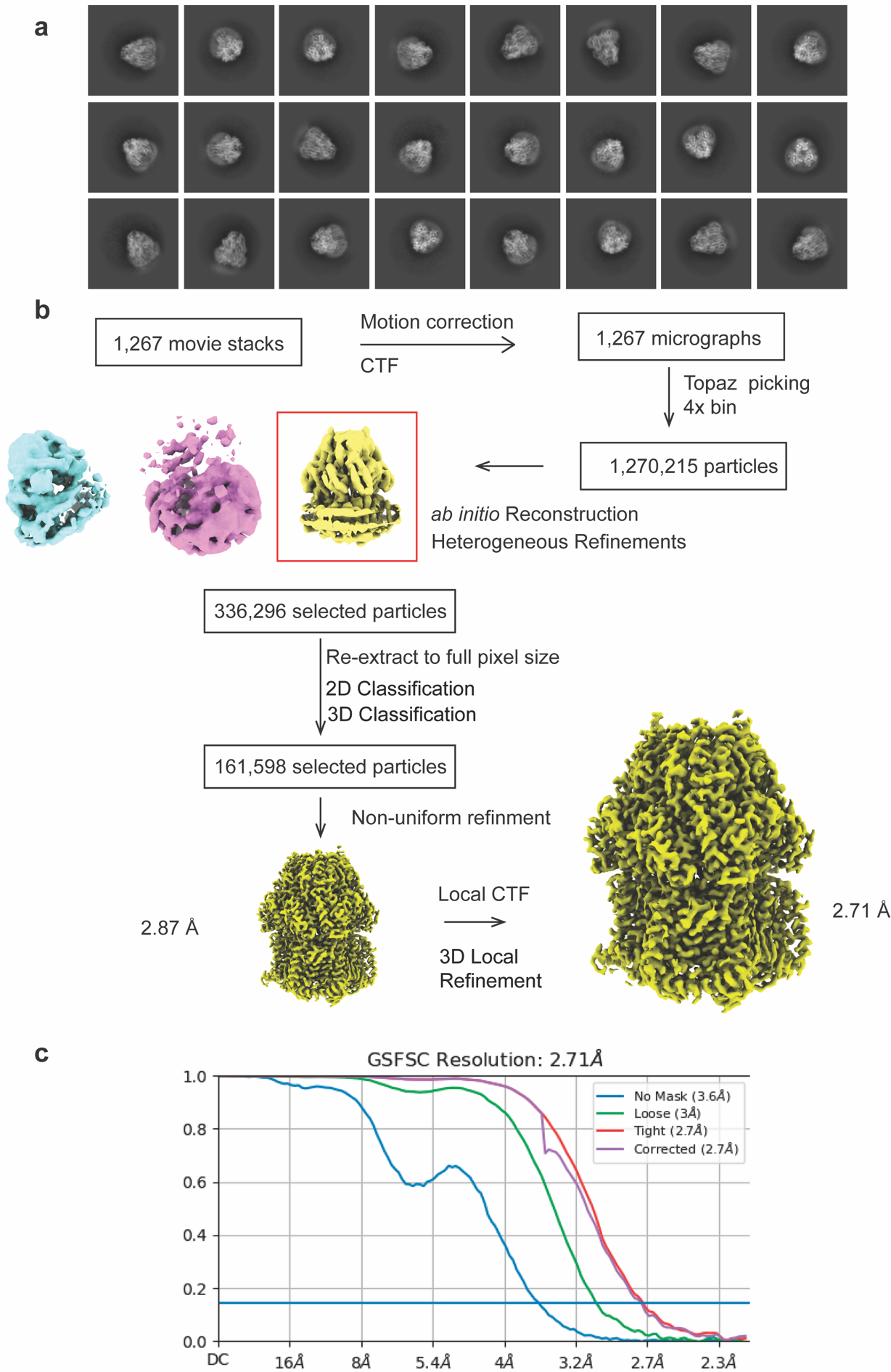
**Figure S3**

**Figure S3. AcrB-CU232 data processing.** **A)** Representative 2D classes of AcrB-CU232. **B)** Data processing workflow of AcrB-CU232. The side view density map of AcrB-CU232 is colored yellow. (c) Gold-Standard Fourier shell correlation (GS-FSC) curves of AcrB-CU232, showing final resolution of 2.71 Å.


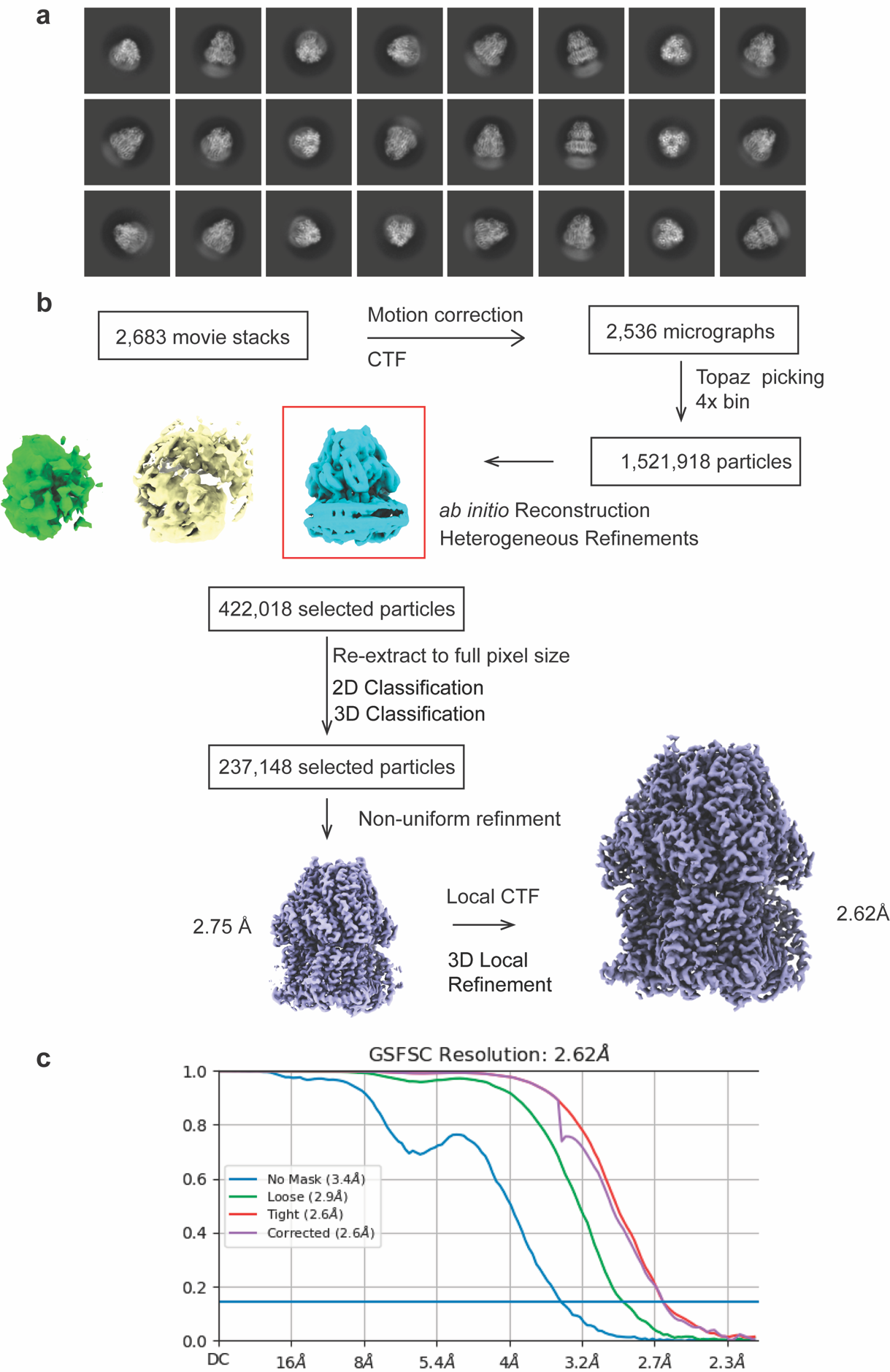
**Figure S4**

**Figure S4. AcrB-CU032 data processing.** **A)** Representative 2D classes of AcrB-CU032. **B)** Data processing workflow of AcrB-CU032. The side view density map of AcrB-CU032 is colored slate. (c) Gold-Standard Fourier shell correlation (GS-FSC) curves of AcrB-CU032, showing final resolution of 2.62 Å.


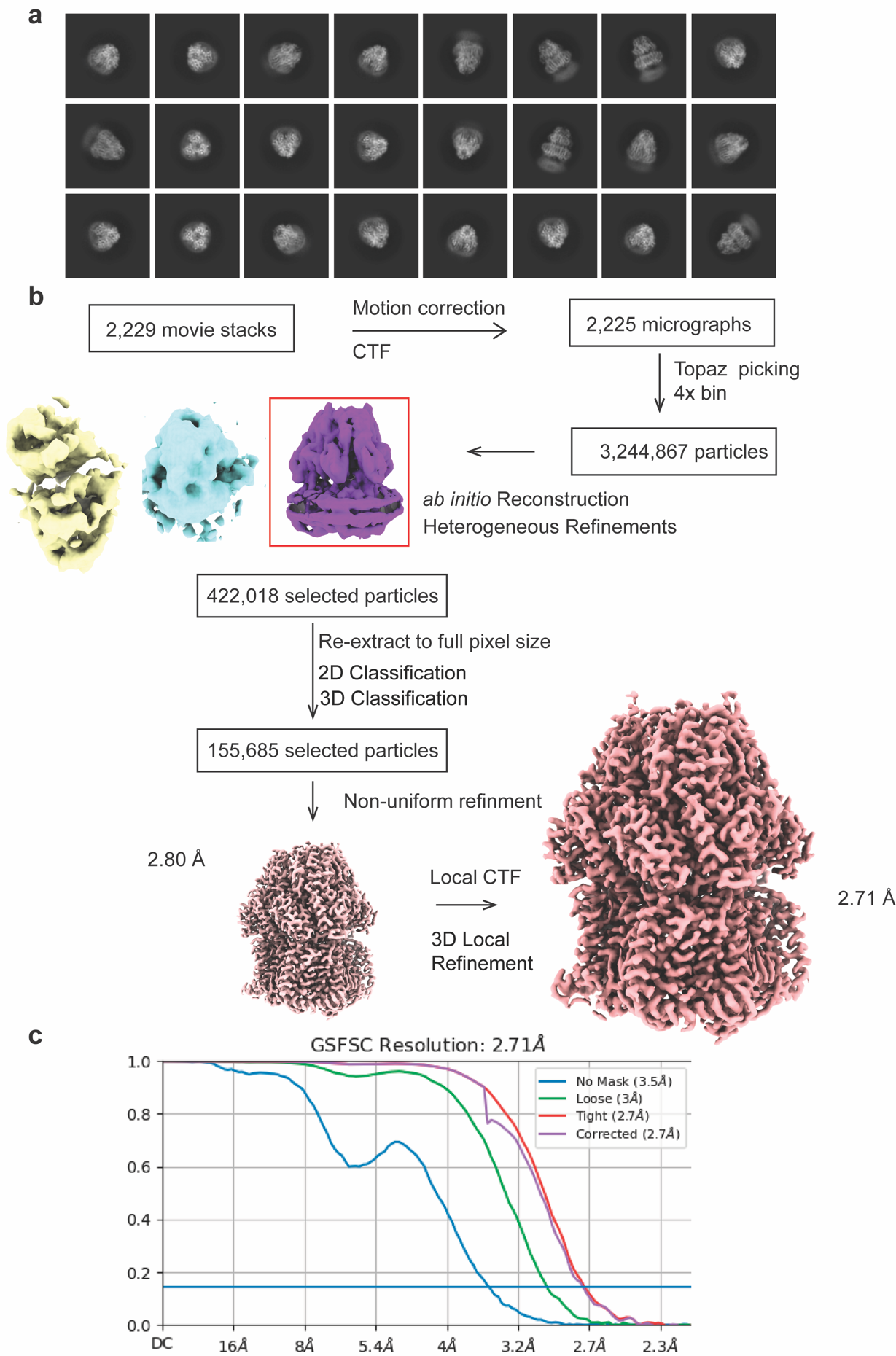
**Figure S5**

**Figure S5. AcrB-EPM35 data processing.** **A)** Representative 2D classes of AcrB-EPM35. **B)** Data processing workflow of AcrB-CU035. The side view density map of AcrB-EPM35 is colored pink. (c) Gold-Standard Fourier shell correlation (GS-FSC) curves of AcrB-EPM35, showing final resolution of 2.71 Å.


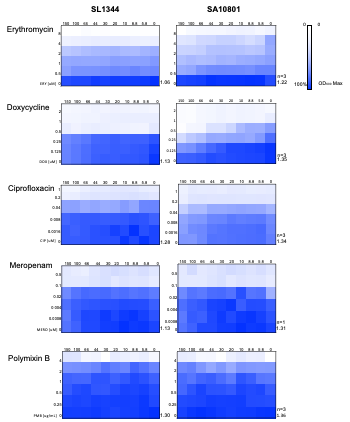
**Figure S6**

**Figure S6. CU032 did not inhibit the growth of *S. enterica* in cation-adjusted MHB.** Checkerboard Assays with CU032 and four classes of clinical antibiotics or the antimicrobial peptide polymyxin B. Two virulent *S. enterica* clinical isolates (S1344 and SA10801) are shown.
